# Feasibility and acceptability of bubble continuous positive airway pressure oxygen therapy for the treatment of childhood severe pneumonia and hypoxaemia in Bangladeshi children

**DOI:** 10.7189/jogh.13.04040

**Published:** 2023-05-26

**Authors:** Mohammod Jobayer Chisti, Trevor Duke, Ahmed Ehnasur Rahman, Tahmeed Ahmed, Shams E Arifeen, John D Clemens, Md F Uddin, Abu SMMH Rahman, Md M Rahman, Tapash K Sarker, SM N Uddin, KM Shahunja, Abu SMSB Shahid, ASG Faruque, Supriya Sarkar, Md Jahurul Islam, Muhammad Shariful Islam, Md Farhad Kabir, Kathrin M Cresswell, John Norrie, Aziz Sheikh, Harry Campbell, Harish Nair, Steve Cunningham

**Affiliations:** 1 International Centre for Diarrhoeal Disease Research, Bangladesh (icddr,b), Dhaka, Bangladesh; 2 Centre for International Child Health, Royal Children`s Hospital, The University of Melbourne, Melbourne, Australia; 3 International Vaccine Institute, Seoul, Korea; 4 UCLA Fielding School of Public Health, Los Angeles; 5 Institute of Child and Mother Health (ICMH), Matuail Dhaka, Bangladesh; 6 250 bedded General Hospital, Kushtia, Bangladesh; 7 University of Queensland, Australia; 8 Directorate General of Health Services, Ministry of Health and Family Welfare, Dhaka, Bangladesh; 9 NIHR Global Health Research Unit on Respiratory Health (RESPIRE), Usher Institute, The University of Edinburgh, Edinburgh, UK

## Abstract

**Background:**

Effective management of hypoxaemia is key to reducing pneumonia deaths in children. In an intensive care setting within a tertiary hospital in Bangladesh, bubble continuous positive airway pressure (bCPAP) oxygen therapy was beneficial in reducing deaths in this population. To inform a future trial, we investigated the feasibility of introducing bCPAP in this population in non-tertiary/district hospitals in Bangladesh.

**Methods:**

We conducted a qualitative assessment using a descriptive phenomenological approach to understand the structural and functional capacity of the non-tertiary hospitals (Institute of Child and Mother Health and Kushtia General Hospital) for the clinical use of bCPAP. We conducted interviews and focus group discussions (23 nurses, seven physicians, 14 parents). We retrospectively (12 months) and prospectively (three months) measured the prevalence of severe pneumonia and hypoxaemia in children attending the two study sites. For the feasibility phase, we enrolled 20 patients with severe pneumonia (age two to 24 months) to receive bCPAP, putting in place safeguards to identify risk.

**Results:**

Retrospectively, while 747 of 3012 (24.8%) children had a diagnosis of severe pneumonia, no pulse oxygen saturation information was available. Of 3008 children prospectively assessed with pulse oximetry when attending the two sites, 81 (3.7%) had severe pneumonia and hypoxaemia. The main structural challenges to implementation were the inadequate number of pulse oximeters, lack of power generator backup, high patient load with an inadequate number of hospital staff, and inadequate and non-functioning oxygen flow meters. Functional challenges were the rapid turnover of trained clinicians in the hospitals, limited post-admission routine care for in-patients by hospital clinicians due to their extreme workload (particularly after official hours). The study implemented a minimum of four hourly clinical reviews and provided oxygen concentrators (with backup oxygen cylinders), and automatic power generator backup. Twenty children with a mean age of 6.7 (standard deviation (SD) = 5.0)) months with severe pneumonia and hypoxaemia (median (md) SpO_2_ = 87% in room air, interquartile range (IQR) = 85-88)) with cough (100%) and severe respiratory difficulties (100%) received bCPAP oxygen therapy for a median of 16 hours (IQR = 6-16). There were no treatment failures or deaths.

**Conclusions:**

Implementation of low-cost bCPAP oxygen therapy is feasible in non-tertiary/district hospitals when additional training and resources are allocated.

Pneumonia is a leading global health problem, accounting for around 740 000 deaths in children under five years of age annually [1], most of which occur in the first two years of life [2]. Hypoxaemia, defined as SpO_2_<90% in peripheral blood on room air [3] is one of the main risk factors for death due to pneumonia among children [4]. Hypoxaemia has been observed in 13% of children requiring hospitalisation for severe and very severe pneumonia classified by the World Health Organization (WHO) in 2005 [5]. However, a recent systematic review revealed the prevalence of hypoxaemia was 31% among all children with pneumonia [6], as classified by the WHO in 2013 [3]. Children hospitalised for severe pneumonia and hypoxaemia in economically-developing countries who received oxygen therapy delivered by low flow (LF) nasal cannula were found to have a 35% reduction in mortality [7] compared to historical controls managed on room air. However, LF oxygen therapy alone is insufficient for many children with severe pneumonia: the case fatality rate (CFR) of hospitalised children receiving LF oxygen for severe pneumonia and hypoxaemia was 23-fold higher (odds ratio (OR) = 22.7, 95% confidence interval (CI) = 6.0-86.0)) compared to children who did not have hypoxaemia [8]. Thus, effective management of hypoxaemia in children with severe pneumonia involving improved respiratory support could contribute to reductions in mortality.

Improved respiratory support for children with severe pneumonia in low-resource settings must be affordable and sustainable. This may include continuous positive airway pressure (CPAP), where an air/oxygen mix is delivered at a pressure that can maintain airway patency. CPAP can recruit the lost volume in pneumonic alveoli, improve functional residual capacity, reduce respiratory rate, improve airway clearance, make breathing more comfortable, and consequently, improve ventilation. It can be delivered via face mask or nasal prongs by mechanical ventilator (MV), machine driven flow driver, or with bubble continuous positive airway pressure (bCPAP), which is comparatively a lower cost method and more easily maintained [9]. bCPAP was successfully used in newborn respiratory distress syndrome both in developed and developing countries [10]. We reported the first successful trial of bCPAP for children beyond the neonatal period with severe pneumonia and hypoxaemia in a developing country [11]. Subsequent trials of CPAP in non-tertiary care settings in Ghana (machine driver CPAP) and Malawi (bCPAP) have shown equivocal or negative results [12,13]. However, in a post-hoc analysis of a Ghana trial, children under one year of age receiving CPAP were found to have better survival [12].

In Bangladesh, district hospitals are secondary-level referral hospitals with 150-250 beds, providing care of paediatric pneumonia patients in paediatric wards, where the limited availability of paediatricians, junior doctors, and nurses limits opportunities for clinical reviews and monitoring. Treatment of pneumonia is limited to antibiotics and LF oxygen, and monitoring equipment (i.e. SpO_2_) is uncommon. As previous CPAP trials demonstrating survival benefit in children with pneumonia were done in tertiary hospitals in intensive care units (ICUs) (Bangladesh) or emergency facilities (Ghana) where paediatricians and/or paediatric intensivist and expert ICU nurses with frequent monitoring opportunities were available, we aimed to explore the gaps and barriers to introducing bCPAP to children with severe pneumonia in district hospitals within a Bangladesh setting. This knowledge would be used to deliver larger implementation through a safety and effectiveness trial in this setting.

We had four objectives: to understand the structural capacity of district hospitals that provide care for children with severe pneumonia, and how those structures might be optimised to ensure trial success; to understand the functional capacity of district hospitals that provide care for children with severe pneumonia and it might be optimised to ensure trial success; to estimate prevalence, treatment failure and deaths of children with severe pneumonia and hypoxaemia before the introduction of bCPAP in two study sites to inform trial power; and to introduce bCPAP to two district hospitals providing care for children with severe pneumonia and hypoxaemia, utilising the knowledge and training materials developed in the first two objectives to understand early clinical safety and adjustments needed for a larger-scale intervention.

## METHODS

### Study design

We used a mixed-methods approach to understand the systems required and potential barriers for delivering bCPAP to children with severe pneumonia and hypoxaemia in district general hospitals in Bangladesh as part of a future implementation study of bCPAP. This study had three pre-treatment groups and one treatment group. One group included the staff (medical and nursing) who provided care to children with severe pneumonia attending and admitted to the participating hospitals and consenting to participate in the study. There were two pre-treatment groups of children who attended the hospitals in the previous 12 months with a clinical diagnosis of pneumonia/severe pneumonia with known outcomes and attended the hospitals in the next three months with clinical pneumonia/severe pneumonia and/or hypoxaemia. One treatment group included children who were prospectively followed up and identified as having severe pneumonia with hypoxaemia and were eligible for bCPAP; we obtained informed written consent from their legal guardians prior to any study procedures.

### Qualitative evaluation

We conducted a qualitative evaluation using a descriptive phenomenological approach [1,2] to understand the structural and functional capacity (**Table 1**) of the non-tertiary hospitals and operational challenges for the introduction of bCPAP. The follow-up assessment was conducted through a participatory approach by actively engaging all participants related to the bCPAP oxygen therapy including consumers or beneficiaries (caregivers – parents or guardians) and service providers (physicians and nurses) in order to fill the gaps in available information and provide different perspectives on complex, contextual, and multi-dimensional phenomena.

**Table 1:** Operational definitions

Parameters	Definitions
Structural capacity	This referred to an assessment of the core estate services that would be required to support the successful delivery of bCPAP that included availability of oxygen source and sterilization facilities of oxygen sources.
Functional capacity	This referred to the skill of the providers to successfully deliver bCPAP that includes staffing levels, training and staff perceptions of technology.
Bubble CPAP oxygen therapy system	It is a combination of three locally available devices: nasal canula, IV infusion set, and plastic bottle. It is prepared by combining one part with another. It is low cost, locally available and easy to prepare.*

bCPAP – bubble continuous positive airway pressure, IV – intravenous

**Figure 1:**
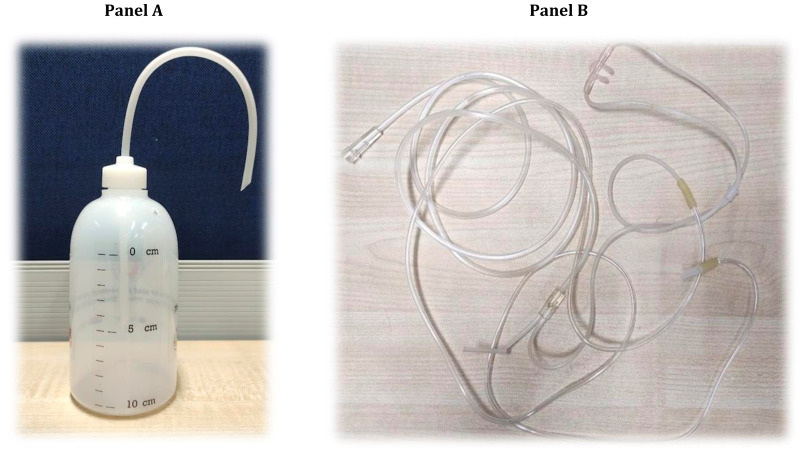
**Panel A. **Bubble CPAP bottle. **Panel B**. circuit system.

*For more details, see Appendix A in the **Online Supplementary Document** and **Figure 1**.

### Quantitative evaluation

We conducted a quantitative assessment of prevalence, treatment failure, and deaths of children with pneumonia and severe pneumonia with or without hypoxaemia in two non-tertiary hospitals before the introduction of bCPAP there. For the children receiving treatment using bCPAP, we followed them up during hospital course and evaluated the treatment failure and deaths in two district hospitals.

### Study settings

We conducted this study in Kushtia General Hospital (KGH) and Institute of Child and Mother Health (ICMH), Matuail Hospital. Both non-tertiary hospitals were located outside of Dhaka, the capital of Bangladesh. The standard care for children under two years of age with severe pneumonia and hypoxaemia in these hospitals was LF oxygen therapy. Both hospitals had one paediatric ward run by qualified physicians and nurses supervised by paediatricians. Those who received bCPAP oxygen therapy were treated in a dedicated corner under nursing vigilance supervised by physicians in the paediatric wards. Children were also treated with intravenous (IV) antibiotics, maintenance fluids, and other supportive care. Patients with severe comorbidities were referred to other nearest tertiary hospitals.

### Eligibility and selection of study participants

#### Qualitative evaluation

Interviewers who were engaged for the qualitative analysis had a social science academic background and were experienced in collecting qualitative data locally. They were trained by a qualified anthropologist. They conducted interviews with clinicians in the hospitals at a place and time convenient for the participants, and with doctors and nurses at hospitals before and after the introduction bCPAP oxygen therapy. They also conducted in-depth interviews at households of patients’ caregivers after the patients received bCPAP oxygen therapy and were discharged from study hospitals. The average duration of an interview was 47 minutes (standard deviation (SD) = 7.6). Interviews were conducted using flexible semi-structured data collection guidelines (Appendix B in the **Online Supplementary Document**) and recorded by audio-recorders. We prepared interview guidelines based on the study objectives and pilot-tested them. We continued data collection until we achieved data saturation.

For this qualitative study, we purposively [1,3] identified 23 participants from ICMH (n = 12) and KGH (n = 11) hospitals before introduction of bCPAP and 21 participants from ICMH (n = 11) and KGH (n = 10) after the introduction of bCPAP to conduct in-depth interviews (IDIs) and focus group discussion (FGDs) (**Table 2**). We purposively selected clinicians who were involved in the study and had been working in the paediatrics department for at least six months. These participants were closely involved in the care of children with severe pneumonia and hypoxaemia receiving bCPAP. We selected different caregivers and influential family members based on their involvement in continued patient care, education level, and age groups of children in their care.

**Table 2:** Methods and study participants

Methods	Before introduction of bCPAP	After introduction of bCPAP
**IDI**	10 IDIs with hospital clinicians	7 IDIs with clinicians
	-	14 IDIs with caregivers
**FGD**	2 FGDs with 13 hospital clinicians	-
n of participants	23	21
Total	44	

IDI – in-depth interview, FGD – focus group discussion, bCPAP – bubble continuous positive airway pressure

#### Quantitative evaluation

For the evaluation of the prevalence of severe pneumonia and hypoxaemia and their outcomes, we included children were aged between two months and 24 months and attended one of the two study hospitals for the assessment of severe pneumonia by hospital clinician. We collected data retrospectively from 1 January 2020 to 31 December 2020 from hospital records and prospectively from 1 March 2021 to 31 May 2021 from the emergency and outpatient departments.

Among children receiving bCPAP, we included those aged between two months and 24 months and diagnosed severe pneumonia with hypoxaemia by a hospital clinician. Parents/guardians gave informed consent to participate in the study. We enrolled the first participant on 21 September 2021 at ICMH and on 29 September 2021 at KGH; both enrolment periods were completed in February 2022.

### Procedure of introduction of bCPAP in patients with severe pneumonia and hypoxaemia

Before the start of screening and enrolment, the study staff organized a training session for the hospital medical staff (physicians, nurses) at both sites. The treatment algorithm, management of severe pneumonia patients, hands-on training, and qualitative and quantitative data management were discussed. The screening started at the ICMH on 12 September 2021 and on 28 September 2021 at KGH. The total feasibility sample size was 20 (10 in each hospital). We screened all patients aged between two months and 24 months admitted in the paediatric ward of both study sites. Only the patients diagnosed with severe pneumonia with hypoxaemia, as diagnosed by hospital physicians, were eligible for enrolment. After confirmation of the diagnosis and subsequent written informed consent from the parents or caregiver, bCPAP was introduced immediately with 5L/min oxygen flow and five centimetres of positive end expiratory pressure (PEEP). The consent procedure was discussed with the parents and all their questions were answered. The participants were followed-up one hour prior to and four hours after the enrolment time point. Staff recorded the patients’ vital observations before the start of bCPAP oxygen therapy. Study staff recorded the baseline information sheet within one hour of enrolment and conclusion form on the day of discharge. Clinical findings, anthropometric examination, and regularly scheduled follow-ups were recorded with the help of attending hospital physicians and nurses. Site investigators reviewed the clinical record forms (CRFs) within 24 hours of completion.

### Data analysis

#### Qualitative evaluation

We transcribed the qualitative interviews verbatim and wrote up observation notes into detailed notes in local language just after fieldwork was completed, after which two researchers coded each transcript and observation notes, compared results, and resolved any discrepancies to ensure trustworthiness of the coding process. We used a thematic coding approach (according to our study objectives) and a framework emerged from interviews and observational data, in which the thematic coding enriched our narrative analysis. We analysed data manually, using both thematic and narrative approaches [1,4], following a sequence of steps [4] that include reading, coding, re-reading, displaying data, reducing, and interpreting textual data using an emic approach [5], after which the main themes were finalised. We presented verbatim the complex issues in the results section.

#### Quantitative evaluation

We analysed the frequency of pneumonia in the population of children aged two to 24 months presenting to our two selected hospitals and the subgroup that had severe pneumonia with and without hypoxaemia. We also analysed dichotomous variables using the χ^2^ test, both prospectively and retrospectively in our selected hospitals. We used the Student’s *t*-test to calculate the mean and SDs for homogenous and non-parametric tests to calculate median and interquartile range for non-homogenous continuous variables. We considered *P* < 0.05 as statistically significant.

### Ethical and governance approvals

The Research Review Committee and Ethical Review Committee of International Centre for Diarrhoeal Disease Research, Bangladesh (icddr,b) (PR-18049) approved the study. We took informed and written consent from the parents of the children and participating doctors and nurses. We also obtained administrated approval from the Ministry of Health to conduct the study in KGH and ICMH.

## RESULTS

### Qualitative results

The structural and functional challenges in introducing bubble CPAP in KGH and ICMH (**Table 3**) include an inadequate number of oxygen cylinder and suction machine, inadequate and non-functioning pulse oximeter, oxygen flow meters and oxygen concentrator, a lack of power generator back-up in paediatric ward and high patient load with an inadequate number of hospital staff especially at KGH. The operational challenges and opportunities disclosed during the clinical use of bubble CPAP oxygen therapy are shown in **Table 4**. The operational challenges included a rapid turnover of hospital-trained clinicians, missing of scheduled routine care of in-patients by hospital clinicians due to their extreme workload (particularly after official hours), lack of adequate bubbling in the water filled plastic bottle especially for mouth breathers. The opportunities were the availability of oxygen concentrators (with back-up oxygen cylinder provided by the investigators) and automatic power generator back-up in ICMH.

**Table 3:** Structural and functional challenges that were identified and provided supports to enable the feasibility study Provided support to enable the feasibility study to go ahead

			Provided support to enable the feasibility study to go ahead		
**Parameters**	**KGH**	**ICMH**	Organisational support*	Supply from study team*	Educational/training*
Pulse oximeter	Only one was available but unable to provide accurate reading of oxygen saturation	Only one was available but non-functioning (damaged battery)	Government supply was not available	Two pulse oximeters/ per hospital	Provided training for maintenance
Oxygen cylinder	Not adequate as one cylinder often needs to deliver oxygen for two patients simultaneously	Adequate	Government supply and ensured for backup in bCPAP treatment corner	No support was required from study team	Provided training for maintenance
Oxygen flow meters	Available but sometimes not properly functioning	Available but sometimes not properly functioning	Government supply	No support was required from study team	Provided operation and maintenance training
Suction machine	Not adequate	Not adequate	Government supply was not available	One machine for KGH as per the request of nursing supervisor	Regular monitoring and cleaning of parts
Central piped oxygen	Available but non-functioning	Available and most of the times are functioning	Government supply was not available	No support was required from study team	Ensured regular monitoring of adequate pressure
Oxygen concentrator	Not adequate and available concentrators are not properly functioning	Adequate and functioning	Government supply was not available	Two concentrators/per hospital	Provided training for operation and maintenance. Ensured regular monitoring and calibration
Medicines	Sometimes shortage in the supply of required medicines (e.g. first line antibiotic)	Sometimes shortage in the supply of required medicines (e.g. first line antibiotic)	Government supply. In case of shortage poor fund was used for some poor patients.	No support was required from study team	-
Electricity	Load shedding occurred frequently especially during the summer season	Load shedding occurred infrequently	Government supply	No support was required from study team	Instructed to use backup oxygen cylinder instead of oxygen concentrator during load shedding
Power generator back-up	Lack of power generator back-up in the pediatric ward	Available, instant generator back-up support	Government supply	No support was required from study team	Instructed to use backup oxygen cylinder instead of oxygen concentrator during load shedding
Number of staff	Not adequate, rapid turnover of trained clinicians	Not adequate particularly during the evening and night	Government is unable to resolve it	No support was required from study team	Refreshers training. Informal training of the trainers. Motivation during enhanced training
Dedicated patients’ bed/treatment corner in front of the nursing station	Initially not available	Available but was not dedicated to patients receiving bCPAP	Respective hospital administration ordered for this arrangement	Motivational support was required from study team	Motivated hospital administration for a dedicated treatment corner in front of nursing station
Bio-engineer to maintain medical equipment	Not available	Not available	Institution is unable to recruit	No support was required from study team	Needs extensive training for the users
Patient load	High patient load	Moderate patents load	Government order (recruitment)	No support was required from study team	Motivation during enhanced training

KGH **–** Kushtia General Hospital, ICMH – Institute of Child & Mother Health, bCPAP – bubble continuous positive airway pressure

*Provided support to enable the feasibility study to go ahead.

**Table 4:** Operational challenges and opportunities observed during the implementation of bCPAP oxygen therapy

Parameters	KGH and ICMH	Provided support to enable the feasibility study to go ahead
Oxygen source: Change of concentrator to cylinder due to power cut	In KGH there was a delay in one case at night because of lack of back-up oxygen cylinder at bCPAP corner. Eventually ensured a backup of oxygen cylinder in bCPAP treatment corner for both hospitals	Motivational education
Availability of arrangement for holding a water-filled plastic bottle	Not available, increased the risk of the water bottle falling down leading to disconnection of nasal cannula and lack of bubbling in the water bottle for a short period	Holder needs to made attached to each bed by the hospital administration
Lack of adequate bubbling in the water filled plastic bottle	Occasional, especially for mouth breather; leaking nasal cannula/prongs inside the nostril; and loose fitting of nasal interface	Mother was educated and motivated to inform nurses in case of lack of bubbling and the nurses found out the problems and fixed them to reproduce the bubbling
Nasal secretion, blockage and dryness of nose	Occasional	Mother was educated and motivated to inform nurses in this situation. Proper cleaning of the nasopharynx using lignocaine-soaked tube adjusted with a suction machine
Experience of doctors and nurses	Although sometimes routine monitoring and follow-ups/reviews of the patients were missed due to high patient load, insufficient clinicians and rapid turn-over of trained staff especially in KGH, bCPAP was found to be simple to handle; no adverse events; rapid disappearance of danger signs with rapid recovery; reduced bed occupancy, Healthcare-associated infections (HAIs) and potential reduced child death, clinicians’ workload; rapid replacement of the beds by newly admitted patients – ultimately reduced hospital cost (food, oxygen & medicines)/per patient	Reinsured
Experiences of caregivers	There was initial anxiety of few mothers due to initial crying, dryness of the mouth & lips of their children; however, eventually all the mothers became happy with the positive outcome (calm, relaxed, breathing comfortably, sleeping, opening of eyes, smiling, improved oxygen saturation, faster disappearance of grunting sound/other danger signs, able to feed, shorter hospital stay) with faster hospital discharge that helped to reduce caregivers’ direct and indirect cost of purchasing food, medicines and traveling to the hospitals	Reinsured

KGH – Kushtia General Hospital, ICMH – Institute of Child and Mother Health, bCPAP – bubble continuous positive airway pressure, HAIs – health care-associated infections

### Quantitative results

#### Retrospectively and prospectively collected data

**Figure 2:**
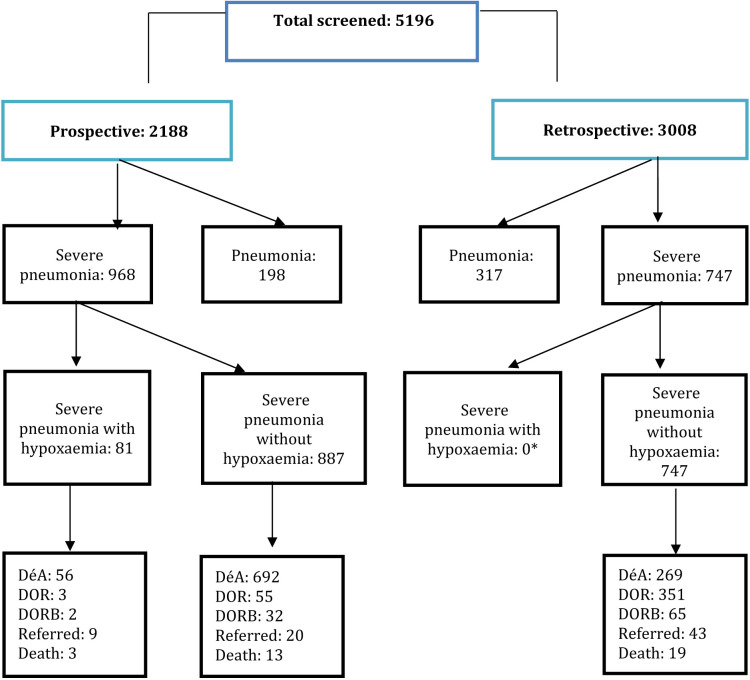
Profile of children with their retrospectively and prospectively collected data. *Not applicable as those were not documented. DéA – discharge with advice, DOR – discharge on request, DORB – discharge on risk bond.

**Figure 2** shows the profile of children with their retrospectively and prospectively collected data. Prospectively collected data showed the frequency of severe pneumonia with and without hypoxaemia. Retrospectively collected data showed the frequency of severe pneumonia with no documentation of hypoxaemia.

The retrospectively collected data in KGH showed that only 1.7% of admitted children over the year had severe hypoxaemia in ICMH and KGH (**Table 5**).

**Table 5:** Prevalence of severe pneumonia with and without hypoxaemia in ICMH and KGH

Diagnosis		KGH (n = 1359), n (%)		ICMH (n = 3837), n (%)	
	**Total (n = 5196), n (%)**	**Prospective (n = 697)**	**Retrospective (n = 662)**	**Prospective (n = 1491)**	**Retrospective (n = 2346)**
Pneumonia	515 (9.9)	53 (7.6)	110 (16.6)	145 (9.7)	207 (8.8)
Severe pneumonia	1634 (31.4)	245 (35.1)	11 (1.7)	642 (43.1)	736 (31.4)
Presence of hypoxaemia	81 (1.6)	41 (5.9)	NA*	40 (2.7)	NA*
Other	2966 (57.1)	358 (51.4)	541 (81.7)	664 (44.5)	1403 (59.8)

KGH – Kushtia General Hospital, ICMH – Institute of Child and Mother Health, NA – not applicable

*Not documented.

The frequency and distribution of clinical characteristics and outcome of children having severe pneumonia with or without hypoxaemia for prospectively and retrospectively collected data are shown in **Table 6** and **Table 7**, respectively.

**Table 6:** Clinical characteristics and outcome of children from prospectively collected data*

Prospective	Total		KGH		ICMH		*P-*value (KGH vs ICMH)	
	**Severe pneumonia with hypoxaemia (n = 81)**	**Severe pneumonia without hypoxaemia (n = 887)**	**Severe pneumonia with hypoxaemia (n = 41)**	**Severe pneumonia without hypoxaemia (n = 245)**	**Severe pneumonia with hypoxaemia (n = 40)**	**Severe pneumonia without hypoxaemia (n = 642)**	**For children with hypoxaemia**	**For children without hypoxaemia**
Age, mean (SD)	8.6 (6.9)	8.2 (5.6)	9.3 (7.5)	10.4 (6.6)	7.9 (6.3)	7.4 (4.9)	0.373	<0.001
Male	45 (55.6)	567 (63.9)	21 (51.2)	146 (59.6)	24 (60)	421 (65.6)	0.427	0.097
Female	36 (44.4)	320 (36.1)	20 (48.8)	99 (40.4)	16 (40)	221 (34.4)	0.427	0.097
DeA	56 (76.7)	692 (85.2)	26 (72.2)	185 (93.4)	30 (81.1)	507 (82.6)	0.371	<0.001
DOR	3 (4.1)	55 (6.8)	0 (0)	0 (0)	3 (8.1)	55 (9)	0.240	<0.001
DORB	2 (2.7)	32 (3.9)	0 (0)	0 (0)	2 (5.4)	32 (5.2)	0.493	<0.001
Referred	9 (12.3)	202 (2.5)	8 (22.2)	12 (6.1)	1 (2.7)	8 (1.3)	0.014	<0.001
Death	3 (4.1)	13 (1.6)	2 (5.6)	1 (0.5)	1 (2.7)	12 (2)	0.615	0.206
Duration of hospital stay in day, median (IQR)	5 (2-7)	4 (3-6)	4 (1-5)	4 (2-5)	6 (5-8)	4 (3-6)	<0.001	<0.001

DéA – discharge with advice, DOR – discharge on request, DORB – discharge on risk bond, SD – standard deviation, KGH – Kushtia General Hospital, ICMH – Institute of Child and Mother Health

*Data are presented as n (%) unless otherwise specified.

**Table 7:** Clinical characteristics and outcome of children from retrospectively collected data*

Prospective	Total	KGH		ICMH			*P-*value (KGH vs ICMH)
	Severe pneumonia with hypoxaemia (n = 0)*	**Severe pneumonia with no documentation of hypoxaemia (n = 747)**	Severe pneumonia with hypoxaemia (n = 0)*	**Severe pneumonia with no documentation of hypoxaemia (n = 11)**	**Severe pneumonia with hypoxaemia, n = 0** **†**	**Severe pneumonia with no documentation of hypoxaemia (n = 736)**	**Severe pneumonia with no documentation of hypoxaemia**
Age, mean (SD)	-	7.6 (5.0)	-	7.55 (5.05)	-	7.61 (5)	0.967
Male	-	494 (66.1)	-	9 (81.8)	-	485 (65.9)	0.349
Female	-	253 (33.9)	-	2 (18.2)	-	251 (34.1)	0.349
DeA	-	269 (36.0)	-	9 (81.8)	-	260 (35.3)	0.001
DOR	-	351 (47.0)	-	0 (0)	-	351 (47.7)	0.001
DORB	-	65 (8.7)	-	0 (0)	-	65 (8.8)	0.612
Referred	-	43 (5.8)	-	2 (18.2)	-	41 (5.6)	0.075
Death	-	19 (2.5)	-	0 (0)	-	19 (2.6)	1.000
Duration of hospital stay in day, median, IQR	-	4 (2-6)	-	3 (3-4)	-	4 (2-6)	0.281

DéA – discharge with advice, DOR – discharge on request, DORB – discharge on risk bond, SD – standard deviation, KGH – Kushtia General Hospital, ICMH – Institute of Child and Mother Health, IQR – interquartile range

†Data are presented as n (%) unless otherwise specified.

#### Introduction of bubble CPAP oxygen therapy

Twenty patients with severe pneumonia and hypoxaemia aged between two and 24 months (md = 4.1, IQR = 3.0-10.3) were enrolled. They had hypoxaemia in room air (md = 87%, IQR = 85-88) with cough (100%) and difficult breathing (100%). Eighteen participants (90%) had a history of fever, 12 (60%) were reluctant to feed, 10 (50%) were lethargic, eight (40%) had grunting respiration, two (5%) had a convulsion, four (10%) had nasal flaring, and three (7.5%) had head nodding (Table S1 in the **Online Supplementary Document**). Four (20%) patients were diagnosed to have concomitant sepsis.

Within an hour of enrolment, the mean (SD) SpO_2_ of all recruited patients was 99% (SD = 1), respiratory rate was 46 (SD = 8) breaths per minute, heart rate was 139 (SD = 31) beats per minute with the support of bCPAP. The average temperature was 36.9°C (SD = 0.7). None of the participants required an increase in oxygen flow and PEEP in the first hour of enrolment. None of the participants required assisted ventilation at any time point of bCPAP oxygen therapy or hospital stay. All the patient’s vitals, including SpO_2_, were recorded every four hours. Pulse oximeters were used to assess the SpO_2_ level. No participants developed hypoxaemia or severe respiratory distress after initiation of bubble CPAP oxygen therapy (**Table 8**).

**Table 8:** Comparison of vital signs between enrolment and after first hour of bCPAP oxygen therapy using paired *t*-test

Parameters	First follow-up (baseline), mean (SD)	Second follow-up (after one hour), mean (SD)	*P*-value
SpO_2_	86.5 (2.5)	98.7 (1.2)	<0.001
Heart rate	149.3 (15.7)	138.5 (30.5)	0.105
Respiratory rate	59.4 (7.7)	45.6 (8.4)	<0.001
Temperature	37.1 (0.8)	36.9 (0.7)	0.176

SpO_2_ – oxygen saturation, SD – standard deviation

All the patients were treated and monitored by physicians and nurses (100%) in a dedicated space in front of the nursing station. An average of 36 (SD = 17) minutes were required to visit and examine the admitted patients by the physicians, increasing to 40 minutes (SD = 24) at KGH. Ten participants (50%) received antibiotics as per the WHO guidelines. None developed treatment failure or any sign of a serious adverse event. One patient (5%) required two episodes of bCPAP oxygen therapy at ICMH.

Two patients (10%) from ICMH were referred to a higher facility – one had hospital acquired infection and another was suspected of having aspiration pneumonia and both the events occurred after terminating the bCPAP oxygen therapy. One participant required an increase in the oxygen flow and PEEP at different time points as the child had grunting respiration (but not due to hypoxaemia) even with the support of 5L/min oxygen and five centimetres water pressure of PEEP. However, the child recovered and was discharged successfully.

The median duration of bubble CPAP oxygen therapy was 16 hours (IQR = 8-16). The median duration of hospital stay was five days (IQR = 4-6) since the patients were discharged after completing the parenteral dose of antibiotics (**Table 9**). There was one reported adverse event that included the discontinuation of bCPAP oxygen therapy due to the stoppage of oxygen concentrator from sudden power cut as there was no back-up oxygen cylinder there at that moment. However, the patient maintained desired oxygen saturation and stayed well.

**Table 9:** Characteristics of study children during hospitalization

Characteristics	Total	ICMH	KGH
Monitored by both physician and nurse, n (%)	20 (100)	10 (100)	10 (100)
Time gap to examination patients by physicians in minutes, mean (SD)	36 (17)	31 ( 3)	40 (24)
**Episodes of bCPAP oxygen therapy, n (%)**			
One episode	19 (95)	9 (90)	10 (100)
Two episodes	1 (5)	1 (10)	0 (0)
**Treatment duration, median (IQR)**			
Duration of bCPAP oxygen therapy	16 (8-16)	16 (8-24)	10 (8-16)
Duration of hospital stay in day	5 (4-6)	6 (5-6)	4.5 (4-7)
**Outcome, n (%)**			
Well and discharged	18 (90)	8 (80)	10 (100)
Referred to superior facility	2 (10)	2 (20)	0 (0)

SD – standard deviation, KGH – Kushtia General Hospital, ICMH – Institute of Child & Mother Health, CPAP – continuous positive airway pressure, bCPAP – bubble continuous positive airway pressure, IQR – interquartile range

## DISCUSSION

Our study identified several important challenges during the assessment of structural and functional capacity of two district hospitals. The main challenges include lack of availability of well-functioning pulse oximeter, oxygen cylinder and flow-meters, oxygen concentrators, adequate number of suction machines, continuous power supply, and a shortage of doctors and nurses in the paediatric ward leading to failure of night follow-ups of some patients receiving bCPAP. The lack of patient reviews, especially at night, was the consequence of the rapid turnover of the trained physicians and nurses in the paediatric ward. During bubble CPAP oxygen therapy, we also identified the lack of holders for keeping water filled plastic bottle and lack of occasional bubbling in the water filled plastic bottle.

Most of these challenges were adequately addressed during the introduction of the clinical use of bubble CPAP oxygen therapy in two non-tertiary hospitals in Bangladesh in order to make the innovative low-cost therapy feasible there. Due to an initial insufficiency and/or non-functionality of medical equipment, the study team solved most of these challenges by making pulse oximeters, oxygen concentrators, suction machine, and nebulisers available before the introduction of bubble CPAP oxygen therapy in both the study hospitals. Next, it was important to ensure four hourly monitoring, with the nurses supervised daily by the qualified doctors. With the available support in view of enhanced training and motivation, hospital doctors evaluated the study patients and, if required, consulted with the paediatrician at least once daily. Notably, both hospitals had paediatricians who were often consulted about the management of study patients. For the instant visibility of the study patients, the investigators ensured the availability of beds in a dedicated space in front of nursing station that ultimately helped routine follow-ups of the patients by the nurses and supervision by the physicians. This arrangement of beds in front of the nursing station was helpful in a hospital with a high patient load as the KGH, which ultimately helped ensure the availability of beds and oxygen therapy, and the continuous monitoring of patients in a crowded environment. The high patient load with missing clinical reviews is common in most of the non-tertiary hospitals in Bangladesh [6,7]. In our study, phone calls of patient attendants for nurses or ward boys (non-clinical supportive staff for the nurses) helped maximise the follow-ups, especially at night. Consequently, the availability of a dedicated space for study patients' beds in both the hospitals in front of nursing station aided in resolving the aforementioned challenges and achieving the expected outcome of the bCPAP oxygen therapy. Our study findings suggest that other similar district hospitals in Bangladesh need to have dedicated treatment spaces in front of nursing station for patients with severe pneumonia and hypoxaemia in order to successfully scale-up of bCPAP oxygen therapy. Additionally, hospital administrators provided back-up oxygen cylinders (in KGH) and a properly functioning central oxygen line (in ICMH) free of cost which contributed almost an uninterrupted oxygen supply to run the bubble CPAP oxygen therapy. Hospitals also provided most of the essential medicines. These practices could be replicated in all district hospitals to enable this level of health care. In a few cases, the Social Welfare Fund in ICMH provided medication assistance to poor patients, but not in KGH. We also had refresher training to overcome the rapid turnover of the trained doctors and nurses in the paediatric ward, as previous studies found doctors and nurses were unable to perform even their routine follow-ups due to a lack of training, particularly in Bangladesh [7,8]. The refresher training may be arranged by a group of well-trained, dedicated, and committed clinicians from the hospital's paediatric ward, which can be very useful in providing training to other clinicians and ward boys of the ward to promote team effort for the effective implementation of bCPAP oxygen therapy [9]. Thus, the lead trainer (training of the trainer) might provide instructions and request clinicians to forward this training to obtain better support during bCPAP implementation. Furthermore, a detailed and simple training manual and standard operation procedures (SOPs) in English and local language (Bangla) on the implementation of bCPAP oxygen therapy and the maintenance of medical equipment must available in the treatment area.

The lack of bubbling in water-filled plastic bottle was resolved by the attending nurses after receiving the information from the parents of the patients. The lack of suitable space to keep the water-filled plastic bottle during the receipt of the bCPAP oxygen therapy can be solved by ensuring a specific secured place or gadget, holder, or stan to keep the bottle safe.

In the quantitative data analysis for KGH, we observed that, retrospectively, 110 of 662 (16.6%) patients had pneumonia and 11 of 662 (1.7%) had severe pneumonia; prospectively, 53 of 697 (7.6%) had pneumonia and 245 of 697 (35%) had severe pneumonia, which is a significant difference in two time points. This observation might be due to several factors. Data were collected retrospectively from January to December 2020. Since April 2020, the anxiety and fear about COVID-19 were high and most of the parents did not visit hospitals with children in their care, either due to fear of COVID-19 and the lockdowns imposed by the Bangladesh government. Moreover, incidence of COVID-19 severe pneumonia in children was significantly lower compared to adults. Furthermore, KGH had to care for COVID-19 adults during the 2020 Wuhan outbreak, and was less focused on treating children. Additionally, the lack of proper documentation of severe pneumonia is a major problem in peripheral district hospitals. However, the remaining data were collected prospectively from March to May 2021; during this period, the two non-tertiary hospitals were focused on providing normal patient care, including the management of paediatric patients. In ICMH, we observed severe pneumonia in 31% of the cases in the retrospectively collected data and 43% in the prospectively collected data; this difference, as well as the one observed for hypoxaemia, might result from the lack of proper documentation on severe pneumonia. Although, COVID-19 related anxiety exists all over the country, ICMH continued to examine their paediatric patients during all over the COVID-19 outbreaks. Thus, we consider that prospectively collected data are optimal to support our future randomised controlled trial (RCT) and implementation research in these hospitals, or in those within other developing countries.

In the quantitative data analysis, after the introduction of the clinical use of bCPAP oxygen therapy in two non-tertiary hospitals, we did not find any serious adverse events or treatment failure or deaths in children with severe pneumonia and hypoxaemia receiving bCPAP oxygen therapy, while 4% of deaths were observed in cases receiving LF oxygen therapy prior to the introduction. This indicates that the bCPAP oxygen therapy is feasible and may be effective if implemented appropriately in non-tertiary hospitals in Bangladesh and other developing countries.

### Strengths and limitations

The main strength of the study is the successful mixed method study design that helped identify practical challenges and potential solutions in introducing the clinical use of bCPAP in non-tertiary hospitals. The main limitation of the study is the lack of adequate background information with seasonal variation of prevalence of severe pneumonia and hypoxaemia due to lack of attaining 12 months prospective data.

### Interpretation

Within existing resources, bCPAP can only be feasible to implement in non-tertiary hospitals in Bangladesh if they are supplied with oxygen concentrators with back-up oxygen cylinder, well-functioning good quality pulse oximeters, suction machines, and enhanced training of the doctors and nurses and a dedicated corner for patients in front of nursing station. However, the extra investment will not be easy without the positive change in the policymakers’ stance regarding the introduction of the clinical use of this life saving innovation.

### Implication for policy, practise, and research

The results of our previously accomplished efficacy trial, followed by this feasibility study in Bangladesh, offers usable information for our future cluster RCT in same type of non-tertiary hospitals in Bangladesh and other developing countries. If the results of the RCT are favourable, and if bCPAP could be scaled-up through a nationwide implementation research in treating childhood severe pneumonia and hypoxaemia, especially in non-tertiary hospitals in Bangladesh and other developing countries, it may potentially help reduce childhood pneumonia-related mortality, further helping in attaining Sustainable Development Goals (SDGs) by 2030.

## CONCLUSIONS

Our study identified several operational challenges for the clinical use of bCPAP oxygen therapy in non-tertiary hospitals in Bangladesh and most of the identified challenges were found to be solvable. After this was mostly addressed, our results suggest innovative locally made low-cost bCPAP oxygen therapy is feasible and may be beneficial if implemented in a dedicated area of a paediatric unit in front of nursing station of non-tertiary hospitals with the daily supervision of physicians.

## Additional material


Online Supplementary Document
