# Supplementary material for: Feasibility and acceptability of bubble continuous positive airway pressure oxygen therapy for the treatment of childhood severe pneumonia and hypoxaemia in Bangladeshi children
Source: J Glob Health. 2023 May 26;13:04040. doi: 10.7189/jogh.13.04040 (PMC10208650; doi:10.7189/jogh.13.04040)
Supplement: Online Supplementary Document [file jogh-13-04040-s001.pdf]

icddr,b	
Title: Standard operating procedure for constructing bubble CPAP	

Document No. 3	Effective date 04-05-2021 Next revision date: 01-01-2021		
	Supersede No: NA	Version No: 1.0	Page | 1 of 5

I. Change/version history

S. No	Version No.:	Effective date	Changes /comments
1	1.0	01/02/2020	New

II. Distribution

**S. No**	**Unit**
1	Institute of Child and Mother Health, Matuail
2	Kushtia 250 bed General Hospital

III. Issue date for current version: 01-02-2020

1. Objective
   This SOP is intended to guide study personnel to properly assemble locally made bubble CPAP.

2. Applicability to and responsibilities of various staff members

**Staff member**	**Responsibility**
Study physician	• Ensure availability of all the necessary materials
Site supervisor/Paediatrician	• Supervise proper assembly & administration of bubble CPAP to trial participants
Paediatrician/ Medical Officer and Study Nurse	• Assemble bubble CPAP using the necessary equipments
Study Nurse	• Administer oxygen using bubble CPAP
Paediatrician/GP and Study Nurse	• First one hour of admission: Close monitoring of the patient, oxygen flow and bubble CPAP device

Staff member	Responsibility
	• There will be 4 hourly follow-ups of study patients after a 1st hour follow-up that include monitoring of the patient, oxygen flow and bubble CPAP device
Study Nurse	• Cleaning of all the connecting parts of bubble CPAP with chlorinated water every 3 or 4 day interval
PI/Study physician	• Refresher training every 6 months interval

3. Required materials and Equipment

Item	Number	Specification
Nasal prong	1	Use infant size for 1-12 months and pediatric size for >12 months
IV fluid tubing	1	None
Graduated water bottle	1	None
Compact 10 Litre Oxygen concentrator	1	Accommodates oxygen tubing upto 50 feet with 7 feet of nasal cannula, Capable of delivering 87% to 96% of O2 purity from 2 to 10 liters per minute (LMP) and outlet pressure 138 kilopascal (kPa) +/- 7 kPa
Hand gloves	1	Disposable
Mask	1	Disposable

4. Step-by-step procedures

4.1 Cut one of the limbs of the nasal prong and tie up the distal end.

4.2 Connect proximal limb of the nasal prong to IV fluid tubing.

4.3 Insert the distal end of the IV fluid tubing to a graduated water bottle.

4.4 Make the depth of the inserted tube into the water at 5 cm. which can be increased up to 8 cm. depending on the child's response.

4.5 Connect the main distal limb of the nasal prong will to oxygen source/ concentrator.

4.6 Check for auto bubbling

N.B Auto bubbling means if the bubbles are produced after initiation of oxygen but before fixing the nasal interface into the nostrils.
If auto bubbling occurs, change the whole circuit and reconstruct again.

4.7 Connect the nasal prong securely to the child's nostrils.

5. Safety measure

The whole device or circuit is hazardous neither to patients nor to the clinical staff. Before the preparation the individual needs to wear gloves. Follow-up every 4 hourly to observe for any obstruction/disconnection/leakage, bubbling, condensate in the expiratory arm in bubble CPAP circuit, nasal trauma/bleeding, abdominal distension or rarely pneumothorax. BCPAP bottle and connecting parts can be reused by cleaning with chlorinated water properly.
Each and every device will be labeled as "this device is to be used for research purposes only" which will prevent other non-specific use. The labeling, perforation and water pressure (in centimeter) marking will be done at the procurement of icddr,b. All the BCPAP bottles will be supplied from icddr,b Dhaka Hospital procurement to each District Hospital. The bottle will be kept in medical equipment containing box or almirah away from sunlight to avoid frequent handling

6. Quality Assurance / Quality Control

All the physicians and study nurses will be trained in this SOP. Refresher trainings will be conducted after every six months for all study staff. The site supervisor also oversees the study personnel when they assemble bubble CPAP.

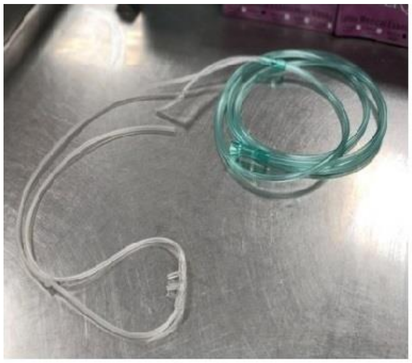

Nasal Canula

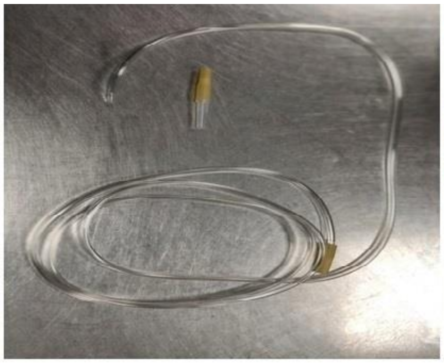

Infusion set

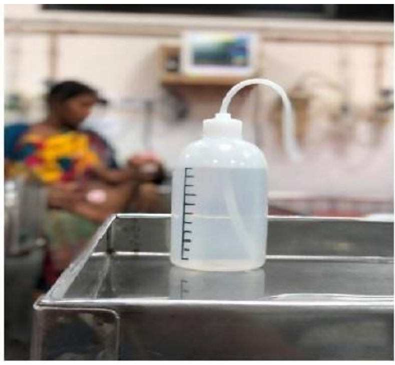

Bottle

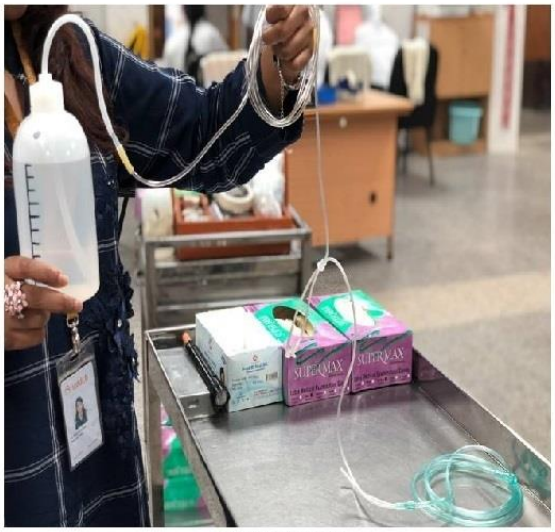

Prepared circuit

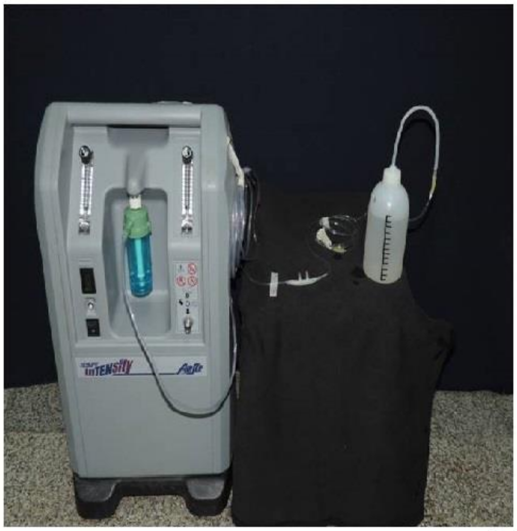

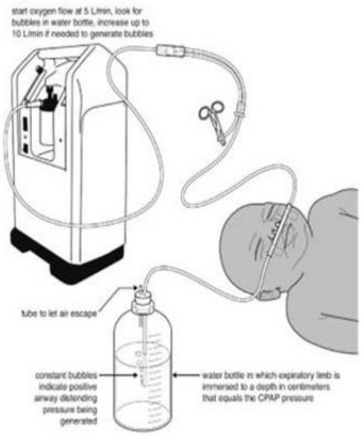

Bubble CPAP

Author :	Reviewer 1st	Approved by:
Name: Dr. Abu Sayem Mirza Md. Hasibur Rahman<br>Designation: Study Physician<br>Signature:__________________	Name: Dr. Abu Sadat Mohammad Sayeem Bin Shahid<br>Designation: Assistant Scientist<br>Signature:________________	Name: Dr. Mohammad Jobayer Chisti<br>Designation: Senior Scientist<br>Signature:______________

Appendix-B

Draft Qualitative Data Collection Guidelines

**Data Collection Guidelines for Feasibility Assessment with Physicians and Nurses (before introduction of bCPAP)**
**For endpoint: Function-human resource**<br>• Is the existing human resource capacity sufficient (existing staff compared to planned) to successfully deliver bubble CPAP in the paediatric ward of the hospital?<br><br>**Specific Questions: Interview with the Hospital Staff (e.g., physician, nurse, medical assistant etc.)**<br>**1. Background information**<br>— Age<br>— Sex<br>— Education<br>— Designation<br>— Years/months of experience<br>— Name of the hospital<br><br>**2. Existing human resources in the paediatric ward**<br>— What categories of staff are currently available in the paediatric ward (e.g., paediatricians, junior doctors, nurses, and clinical officers, attendant etc.)? What are the specific roles and responsibilities of them? Please probe.<br>— Is the workforce sufficient for the paediatric ward (e.g., planned and existing staffing)? Please explain in your own way. What happens if there remain insufficient staffs?<br>— How staffs perform their regular duties? Please probe (e.g., working hours, number of staff available at day/night-who are they etc.) Are they facing any difficulties? How they solve these?<br>**3. Staff for providing treatment of severe pneumonia patients**<br>— Who (e.g., paediatricians, junior doctors, nurses, and clinical officers, attendant etc.) are specifically assigned for providing the current treatment to the severe pneumonia patients? Is the workforce sufficient (e.g., planned and existing staffing)? If yes/no, why? Probe.<br>— What are the duties they perform? How? Please explain the duties of each staff.<br>**4. The capacity of existing human resources for providing treatment of severe pneumonia patients**<br>— Are the assigned staffs able to provide the current treatment for severe pneumonia patients perfectly or as planned (e.g., scheduled follow up, eliminate any chance of any nasal trauma, nasal bleeding, abdominal distension etc.)? If yes, what makes it possible (e.g., favourable working hours, patient volume, availability of staff, job experience, training, the young age of staff, well coordination among staff etc.?<br>o If no, why (e.g.., supposed to supporting other staff or perform additional duties, allowance factors etc.)? What happens then (e.g., takes support from other staff, refer to other facilities)? Is there found any adverse consequences of it or not? Please explain in detail.<br>— What is your suggestion for effective management of serve pneumonia patient in this hospital through the utilization of existing staff? Please explain in detail.
**For endpoint: Function-staff perception**

- What are the perceptions of hospital staff about the importance of the introduction of bubble CPAP and capacity for its effective management?

Specific Questions: Interview with the Hospital Staff (e.g., physician, nurse, medical assistant etc.)

1. **Current treatment practices in paediatric ward for severe pneumonia patients-staff experiences**
   - What kind of treatment (e.g., WHO standard LF oxygen therapy) is currently available for children with pneumonia in this hospital?
     - How to provide the treatment? How the monitoring system has been functioning over the treatment courses? Is there found any difficulty or not? Why? How solved it? Please tell me in detail.
   - Is the patient or provider facing any difficulty while receiving and providing the treatment? Please probe...patient's difficulty in breathing using WHO LF, difficult to manage, staff capacity in relation to skills and knowledge gap, feeling comfort to work or not, obstacles to providing antibiotics and oxygen for children with severe pneumonia within one hour of hospitalization. Why? What were the responses of the clinical staff to the clinical problem? Explain in detail (positive and negative experiences).
   - What are the outcomes of current treatment practices (e.g., improved, deteriorated, refer to other hospitals, death etc.)? Explain in details the reason for each of the condition.
   - How much cost requires providing the current treatment? Who bear it (e.g., child's parents or hospital)? Is it feasible and cost-effective? If yes/no, why?
2. **The perceived need for improved low-cost respiratory support treatment and its management capacity in the district staff**
   - Do you feel the necessity of low cost improved respiratory support using a simple device in this district hospital for treating the severe pneumonia patients despite implementation of WHO standard LF oxygen therapy?
     - If yes/no, why (e.g., often death occur, expected patients volume, available human resources etc.)?
     - What would be the risks and benefits of its introduction within the clinical environment of the hospital?
   - Do you think that the existing human resources have the capacity to operate the low-cost bubble CPAP treatment using a simple device? Why you perceive this? Please explain.
     - Which category staff would be included or best suited for operating the bubble CPAP in the paediatric ward? Why do you think so?
     - How staff capacity will boost or built if we would like to implement bubble CPAP in this hospital (based on previous experiences)? Please probe (e.g., training needs-formal training/in-service training; need more information and other support)?

Qualitative Data Collection Guidelines for Feasibility and Acceptability Assessment (after introduction of bCPAP)

For endpoint:

- What are the perceptions, motivations, and attitudes towards the introduction and uses of bubble CPAP treatment in the hospital?

<u>Guideline for conducting an in-depth interview with hospital staff (Physician/Nurse) and research staff</u>

Background Information

- Age, sex, education, current position
- Affiliated institution/ name of the hospital
- Years/months of experience
- Roles and responsibilities (specific role related to bubble CPAP)

Knowledge about bubble CPAP Treatment

- Do you know about the bubble CPAP treatment? Please tell me in detail.
    - Please probe (e.g., name, comprehensive treatment plan, benefits, and risk, the function of the machine, potential complication and solutions). How did you inform these?
    - What do you think about your knowledge on bubble CPAP treatment sufficient or not? Why do you think so? Please tell me in detail.

Decisions, motivations, and attitudes towards providing the bubble CPAP treatment

- How the decision was made for providing BCPAP treatment to a patient? Tell me in detail (e.g., in which condition of child's illness).
- How you motivate the patient's caregiver or guardian for providing the new treatment?
    - What kinds of information did you provide them? What were their responses or attitudes (e.g., positive OR negative)?
    - Did you face any difficulty to motivate them? If, yes/no, why? If yes, how you motivate them?

Experiences with bubble CPAP treatment practices: perceptions, motivations and attitudes

- How bubble CPAP was introduced? Were you present there? What was your specific responsibilities and observation? Please probe.
    - Did you ever face or observe any difficulty (e.g., prepare the bubble CPAP circuit, handle nasal prong and water bottle, and operate oxygen concentration, unavailability of electricity)? If yes or no, why? What were the consequences? Explain your opinion.
- Did the patient feel significant discomfort/comfort while introducing the BCPAP oxygen therapy? Why do you think so? Was there any comment from patient's guardian or caregiver (good or bad)? Please tell me detail.

- How bubble CPAP was maintained throughout the treatment courses? Did you observe or face any interruption in maintenance of bubble CPAP? Why? If yes, how it was solved?
- What were the opinions or comments or queries and attitudes of patients' caregivers or guardian regarding the bubble CPAP throughout the treatment courses (positive & negative)? Explain in detail.
- What are the outcomes of bubble CPAP practices in your hospital (e.g., improved phenomena and discharged, discharged with medication, died at the hospital etc)? Please explain in detail.
    - Are you satisfied or dissatisfied with the treatment? Please explain, why or why not?
- Can you tell me the effectiveness of bubble CPAP treatment compared to the current treatment practices? Why do you think so (e.g., low cost, easy to operate)? Tell me in detail.

Recommendations

- Is the bubble CPAP treatment can be a part of the standard of care in the hospital? Why?
- What do you think about its suitability and necessity of implementing bubble CPAP in other hospitals in your country? Why? How it can be implemented? Tell me about your opinion.
- Do you have any suggestions that can ensure the quality of bubble CPAP treatment delivery further? Tell me detail.

Guideline for Conducting In-depth Interview with Patient's Caregiver

Background information

- Age (child and caregiver), sex (child and caregiver), marital status, education
- Relationship with the patient, employment status
- Household's income, number of children

Knowledge about bubble CPAP Treatment

- Do you know about the bubble CPAP treatment? Please probe (e.g., name, comprehensive treatment plan, benefits, risk, the function of the machine, potential complication etc.).
    - How did you inform these? When (e.g., before or after given the treatment)?
    - Did you understand clearly the information given to you? If yes/no, why?
    - How was the information beneficial to you? Please probe (e.g., understand the benefits and risks of treatment).

Motivation towards the introduction of bubble CPAP Treatment

- What were the things motivated you for receiving the bubble CPAP treatment?
    - How you made the decision?
    - Did you face any difficulty (e.g., any confusion, de-motivated by other family member, relatives or other)? How you solved this? Please explain in details.
- What was your feeling about the bubble CPAP treatment before making the decision?
    - Did you feel fear, cry, anxiety, worry, disappointment regarding the BCPAP treatment (e.g., related to the perception of pain inflicted on the child, or a possibility of death)? Why?
    - Did you perceive any positive impression about its possible treatment outcome? Why?

Experiences with bubble CPAP treatment practices: perceptions, motivations and attitudes

- How bubble CPAP was introduced to your child? Explain in your own ways.
  - Were you present there? What was your personal observation? Please tell me in details.
  - Was there any difficulty (e.g., faced by your child or the physician, facilities unavailable)? What things made you comfort or discomfort during the introduction of bubble CPAP?
- Did your child feel or response discomfort/comfort while receiving the oxygen therapy treatment? Why you perceived this (e.g., improved child's sickness sign and symptoms)?
- How bubble CPAP was maintained by doctor, nurses and ward attendant? What sorts of services patients' were provided (including the patients follow up)? Were the services sufficient/insufficient?
  - Did you observe any interruption in the maintenance of bubble CPAP? How these were solved?
- What things made you comfort or discomfort throughout the bubble CPAP treatment courses? Why?
  - Please probe (e.g., any mental stress, other children die on that oxygen therapy treatment, improved child's sickness symptoms etc.)
  - Did you face any challenge for handling or caring your child during the treatment?
  - Did you have any questions or concerns to physician or nurses? Were they able to provide you with the best answer to your questions or concerns?
  - How you were overcome or relieved or adapted to this situation (e.g., support by a doctor, nurses, attendant, research staff, family member, other)?
- Can you explain your child health improvement after the introduction of bubble CPAP (e.g., improved phenomena and discharged, discharged with medication, died at the hospital etc)? Please explain in detail.
  - Are you satisfied or dissatisfied with the treatment? Please explain, why or why not?
- Can you tell me the effectiveness of bubble CPAP treatment compared to the other practices? Why do you think so (e.g., low cost, easy to operate and care)? Tell me in detail.

Recommendations

- Do you perceive the necessity of bubble CPAP in treating other severe pneumonia patients? Tell me in detail.
- Do you have any suggestions that can ensure the quality of bubble CPAP treatment delivery further? Tell me detail.

Supplementary table 1: Baseline characteristics of children received bubble CPAP oxygen therapy

Characteristics	Total	ICMH	KGH
**Clinical information**			
Cough (%)	20 (100)	10 (100)	10 (100)
Presence of hypoxemia (%)	20 (100)	10 (100)	10 (100)
Oxygen saturation/SpO2 (Median, IQR)	87 (85,88)	87 (86,88)	87 (85,88)
Fever (%)	18 (90)	10 (100)	8 (80)
Respiratory difficulties (%)	20 (100)	10 (100)	10 (100)
Feeding difficulties (%)	12 (60)	3 (30)	9 (90)
Convulsion (%)	2 (10)	0 (0)	2 (20)
Cyanosis (%)	0 (0)	0 (0)	0 (0)
History of pneumonia last year (%)	4 (20)	3 (30)	1 (10)
Added sound in lungs (%)	17 (85)	7 (70)	10 (10)
No sign of dehydration (%)	20 (100)	10 (100)	10 (100)
Oxygen received prior to hospitalization (%)	3 (15)	0 (0)	3 (30)
Received antibiotic prior to hospitalization (%)	13 (65)	7 (70)	6 (60)
**Mentation/Appearance**			
Well/alert (%)	6 (30)	0 (0)	6 (60)
Lethargic (%)	10 (50)	6 (60)	4 (40)
Irritable (%)	4 (20)	4 (40)	0 (0)
**Vitals**			
Heart rate (Mean±SD)	149±16	144±18	154±12
Respiratory rate (Mean±SD)	59±8	62±6	56±8
Axillary temperature (Mean±SD)	37.1±0.8	37.8±0.7	36.5±0.5
**Respiratory effort**			
Lower chest wall (%)	20 (100)	10 (100)	10 (100)
Grunting (%)	8 (40)	0 (0)	8 (80)
Head nodding (%)	3 (15)	2 (20)	1 (10)
Nasal Flaring (%)	4 (20)	2 (20)	2 (20)
